# Benign clear cell “sugar” tumor of the lung in a patient with Birt-Hogg-Dubé syndrome: a case report

**DOI:** 10.1186/s12881-016-0350-y

**Published:** 2016-11-21

**Authors:** Yoko Gunji-Niitsu, Toshio Kumasaka, Shigehiro Kitamura, Yoshito Hoshika, Takuo Hayashi, Hitoshi Tokuda, Riichiro Morita, Etsuko Kobayashi, Keiko Mitani, Mika Kikkawa, Kazuhisa Takahashi, Kuniaki Seyama

**Affiliations:** 1 Divisions of Respiratory Medicine, Juntendo University Faculty of Medicine and Graduate School of Medicine, 3-1-3, Hongo, Bunkyo-Ku, Tokyo, 113-8431 Japan; 2 Department of Pathology, Japanese Red Cross Medical Center, 4-1-22, Hiroo, Shibuya-Ku, Tokyo, 150-8935 Japan; 3 Departments of Pathology, JCHO Tokyo Yamate Medical Center, 3-22-1 Hyakunin-cho, Shinjuku-Ku, Tokyo, 169-0073 Japan; 4 Human Pathology, Juntendo University Faculty of Medicine and Graduate School of Medicine, 3-1-3, Hongo, Bunkyo-Ku, Tokyo, 113-8431 Japan; 5 Respiratory Medicine, JCHO Tokyo Yamate Medical Center, 3-22-1 Hyakunin-cho, Shinjuku-Ku, Tokyo, 169-0073 Japan; 6 Thoracic Surgery, JCHO Tokyo Yamate Medical Center, 3-22-1 Hyakunin-cho, Shinjuku-Ku, Tokyo, 169-0073 Japan; 7 Laboratory of Proteomics and Biomolecular Science, Biomedical Research Center, Juntendo University Faculty of Medicine and Graduate School of Medicine, 3-1-3, Hongo, Bunkyo-Ku, Tokyo, 113-8431 Japan; 8 The Study Group of Pneumothorax and Cystic Lung Diseases, 4-8-1 Seta, Setagaya-Ku, Tokyo, 158-0095 Japan

**Keywords:** Folliculin, Loss of heterozygosity, Tumor suppressor gene syndrome, Germline mutation

## Abstract

**Background:**

Birt-Hogg-Dubé (BHD) syndrome is a rare inherited autosomal genodermatosis and caused by germline mutation of the *folliculin* (*FLCN*) gene, a tumor suppressor gene of which protein product is involved in mechanistic target of rapamycin (mTOR) signaling pathway regulating cell growth and metabolism. Clinical manifestations in BHD syndrome is characterized by fibrofolliculomas of the skin, pulmonary cysts with or without spontaneous pneumothorax, and renal neoplasms. There has been no pulmonary neoplasm reported in BHD syndrome, although the condition is due to deleterious sequence variants in a tumor suppressor gene. Here we report, for the first time to our knowledge, a patient with BHD syndrome who was complicated with a clear cell “sugar” tumor (CCST) of the lung, a benign tumor belonging to perivascular epithelioid cell tumors (PEComas) with frequent causative relation to *tuberous sclerosis complex 1* (*TSC1*) or *2* (*TSC2*) gene.

**Case presentation:**

In a 38-year-old Asian woman, two well-circumscribed nodules in the left lung and multiple thin-walled, irregularly shaped cysts on the basal and medial area of the lungs were disclosed by chest roentgenogram and computer-assisted tomography (CT) during a preoperative survey for a bilateral faucial tonsillectomy. Analysis of the resected tumor showed large polygonal cells with clear cytoplasm proliferating in a solid pattern. Immunohistochemistry revealed that these tumor cells were positive for microphthalmia-transcription factor, S100, and CD1a but negative for HMB45, indicating that the tumor was a CCST. Genetic testing indicated that the patient had a germline mutation on exon 12 of the *FLCN* gene, i.e., insertion of 7 nucleotides (CCACCCT) (c.1347_1353dupCCACCCT). Direct sequencing of the *FLCN* exon 12 using genomic DNA obtained from her microdissected CCST cells clearly revealed loss of the wild-type *FLCN* sequence, which confirmed complete functional loss of the *FLCN* gene. On the other hand, no loss of heterozygosity around *TCS1*- or *TSC2*-associated genetic region was demonstrated.

**Conclusion:**

To our knowledge, this is the first report of CCST of the lung in a patient with BHDS, indicating that CCST should be added to the spectrum of pulmonary manifestations of BHDS.

## Background

Birt-Hogg-Dubé (BHD) syndrome is a rare inherited autosomal genodermatosis originally reported by Hornstein and Knickenberg in 1975 [1] and by three Canadian doctors (Birt, Hogg, and Dubé) in 1977 [2]. BHD syndrome is caused by mutations of the *FLCN* gene, which codes a protein called folliculin and functions as a tumor suppressor gene [3, 4]. Several tumor-suppressor genes are linked to hamartoma syndromes through the convergent energy/nutrient-sensing pathways involved in the mechanistic target of rapamycin (mTOR) signaling, especially mTOR complex-1 (mTORC1) [5–7]: *TSC1* and *TSC2* being responsible for tuberous sclerosis complex (TSC) [8], *LKB1* for Peutz-Jeghers syndrome [9], and *PTEN* for Cowden syndrome [10]. Since mTORC1 is strongly related to cell growth, proliferation, and metabolism, hamartoma syndromes are characterized by an increased risk of the development of benign as well as malignant neoplasms. BHD syndrome is one such hamartoma syndrome. Recent studies have shown the close association of folliculin with the mTORC1 signaling pathway; folliculin acts as a GTPase-activating-protein for RagC/D to recruit mTORC1 to lysosomes where mTORC1 exerts its function [11]. Accordingly, patients with BHD syndrome are at risk of developing neoplasms in skin (fibrofolliculoma) and kidneys (renal cell carcinoma), colon carcinoma, or other tumors. However, no pulmonary tumor associated with BHD syndrome has been reported to date.

Clear cell “sugar” tumor (CCST) of the lung is a rare benign tumor originally reported in 1971 by Liebow and Castleman, who noted its resemblance to metastases of clear renal cell carcinomas [12]. CCSTs belong to a family of perivascular epithelioid cell tumors (PEComas) that are defined by their morphologically and immunohistochemically distinctive perivascular epithelioid vascular cells and arise at a variety of visceral and soft tissue sites [13]. PEComas include angiomyolipomas [14] and lymphangioleiomyomatosis [15], both being the representative disorders related to TSC. Actually, the genetic and molecular bases of PEComas appear to be aberrations on chromosome 16 and at the *TSC2* locus resulting in activation of the mTOR pathway [16].

We herein describe a patient with BHD syndrome who developed CCST of the lung. We demonstrated that this patient’s CCST entailed complete functional loss of *FLCN* without *TSC2* loss of heterozygosity (LOH), supporting the presumption that *TSC1/2* and *FLCN* share the mechanism for their tumorigenesis.

## Case presentation

**Fig. 1:**
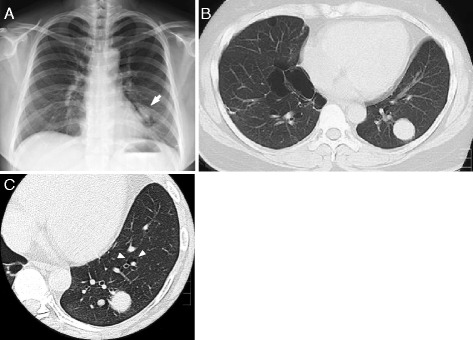
Plain radiograph and computed tomography of the chest. Plain chest radiograph on admission showed a nodular shadow superimposed on the edge of a cardiac silhouette (**a**). Computed tomography of the chest demonstrated a round nodule (the larger nodule, 25 mm in diameter) with a clear margin in the lower, left lobe and multiple irregularly shaped cysts in the bilateral lower lobes (**b**). Another axial image showed a smaller nodule (5 mm in diameter) having a similar characteristics with the larger nodule (**c**). Note that a small cyst abutting a pulmonary artery (*white arrowheads*)

**Fig. 2:**
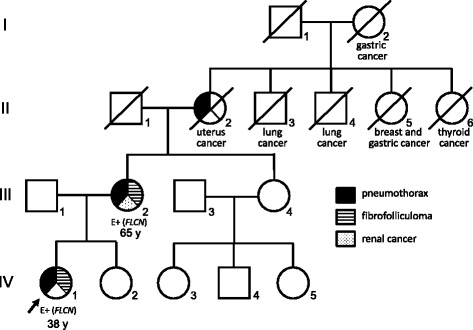
The family pedigree. The family pedigree includes a symbolic presentation of the three clinical features of Birt-Hogg-Dubé (BHD) syndrome: pneumothorax, fibrofolliculoma-like papules (not proven by biopsy), and renal cancer. The Roman numerals at the left side represent generations. *FLCN* genetic testing was performed in the index case (IV-1, arrow) and her mother (III-2), and both were demonstrated to carry the germline *FLCN* mutation [indicated by E+(*FLCN*)]. A marked prevalence of cancer-related death was found among maternal first- and second-generation descendants. The index case and mother also had a past medical history of malignancy (as described in the text)

A solitary pulmonary nodule measuring 25 mm in diameter was found in the left lung of a 38-year-old female whose chest roentgenogram (Fig. 1a) underwent preoperative assessment in January 2006 during preparation for a bilateral faucial tonsillectomy due to recurrent tonsillitis. The computer-assisted tomography (CT) of the chest showed two well-circumscribed nodules (approximately 25 and 5 mm in diameter, respectively) without apparent contrast enhancement in the lower, left lobe (Fig. 1b and c). In addition to the nodules, multiple thin-walled, irregularly shaped cysts were distributed predominantly on the basal and medial areas of the lungs (Fig. 1b and c). She was asymptomatic, and physical examinations found no abnormalities. She was an ex-smoker but to the very mild degree of 0.6 pack-year. However, her past medical history was remarkable. Vocal cord nodules were resected when she was 25-, 26-, and 27-years-old; three episodes of spontaneous pneumothorax (PTX) occurred at the ages of 26, 27, and 30 years (chest tube drainage for left PTX at 26-years-old, and operations were performed for a right PTX at 27- and for left PTX at 30-years-old). The resection of multiple uterine myomas followed when she reached 30 years of age, and the left-sided thyroid gland was extirpated to remove a well-differentiated papillary adenocarcinoma when she was 32-years-old. The family history taken from interviews with the patient and her mother was extraordinary in terms of PTX events and neoplasms (Fig. 2). Her mother (III-2 in Fig. 2) had experienced spontaneous PTX and renal cancer (no detailed information on histological type), and the maternal grandmother (II-2 in Fig. 2) had undergone a spontaneous PTX. Fibrofollicuoma-like papules were noted on faces and necks of both the patient (IV-1 in Fig. 2) and her mother, but were not mentioned for any other family members during their interviews. Later, we confirmed by CT of the chest that the mother had multiple pulmonary cysts with characteristic features similar to those of the index case.

For the patient described here, a CT-guided needle biopsy of the left pulmonary nodule (the larger nodule) was performed to determine a diagnosis. Pathological examination revealed that it was a clear cell carcinoma of undetermined origin. No clear cell carcinoma of the kidney was identified by magnetic resonance imaging. Ultrasonography of the thyroid gland showed no abnormalities. Subsequently, on February 28, 2006, a left lower lobectomy was performed. The left hilar lymph node was negative for metastasis, and no further resection of mediastinal lymph nodes was performed. Neither recurrence nor metastasis has been found since then or up to the writing of this case report in 2015.

## Materials and methods

### Histopathologic and immunohistochemical examinations

The patient’s resected lung tissues were fixed in 10% buffered formalin, embedded in paraffin after routine processing, and stained with hematoxylin and eosin (H&E). Immunostaining was performed with antibodies directed to the following: vimentin (dilution 1:400, Dako Cytomation, Carpinteria, CA, USA), cytokeratin (dilution 1:200, Immunotech, Malseille, France), α-smooth muscle actin (α-SMA) (dilution 1:200, Dako Cytomation), HMB45 (dilution 1:200, Dako Cytomation), Melan-A (dilution 1:50, Dako Cytomation), PNL-2 (dilution 1:200, Dako Cytomation), microphthalmia-associated transcription factor (dilution 1:50, Leica Biosystems, New Castle, UK), S100 (dilution 1:300, Dako Cytomation), CD1a (dilution 1:100, Dako Cytomation, clone O10), CD10 (dilution 1:100, Dako Cytomation), CD63 (dilution 1:400, Chemicon, Temecula, CA, USA), and folliculin (dilution 1:150, Santa Cruz, Paso Robles, CA, USA). An EnVision Dual LINK system-HRP (a polymer-based detection system, Dako Cytomation) was used to detect binding of the first antibodies according to the manufacturer’s instructions, and 3, 3′-diaminobenzidine tetrahydrochloride was used as the chromogen.

### Mutation analysis of the *FLCN* gene and LOH analysis of the *FLCN* gene-associated region

Genomic DNA isolated from peripheral blood leukocytes was utilized to identify a germline *FLCN* mutation according to the method described previously [17]. Briefly, each exon of the *FLCN* gene was separately amplified and then screened by denaturing high-performance liquid chromatography. When a mobility shift was detected, sequencing of the exon of concern was performed using an automated sequencer (ABI Prism BigDye Terminator v1.1 Cycle Sequencing kit and ABI 3130 Genetic Analyzer; both Applied Biosystems, Foster City, CA, USA). Cloning of the PCR product was performed using a TA cloning kit (Invitrogen ^TM^, Houston, TX, USA).

For LOH analysis of the *FLCN* gene-associated region (chromosome 17p12.2), tumor cells in a lung nodule were manually microdissected from an 8-μm-thick pathological specimen under an inverted microscope. Normal alveolar tissues were also microdissected as a control. Genomic DNA was extracted from the microdissected cells with 25 μl extraction buffer consisting of 50 mM Tris–HCl (pH8.0), 1 mM EDTA, 0.5% Tween 20, and 1/50 volume of proteinase K solution (10 mg/ml). Two microsatellite markers, *D17S740* and *D17S2196*, were examined for LOH analysis according to the method described by Khoo et al. [18]; the *FLCN* gene is located on the genome between these two markers. The PCR products were electrophoresed using an Applied Biosystems 3130/3130xl Genetic Analyzer. LOH analysis was performed using Gene Mapper® 4.0 (both from Applied Biosystems, Foster City, CA, USA). To confirm reproducibility, all lesions were examined at least twice. A reduction in signal intensity over 50% was defined as LOH.

## Results

### Pathologic findings in pulmonary nodules

**Fig. 3:**
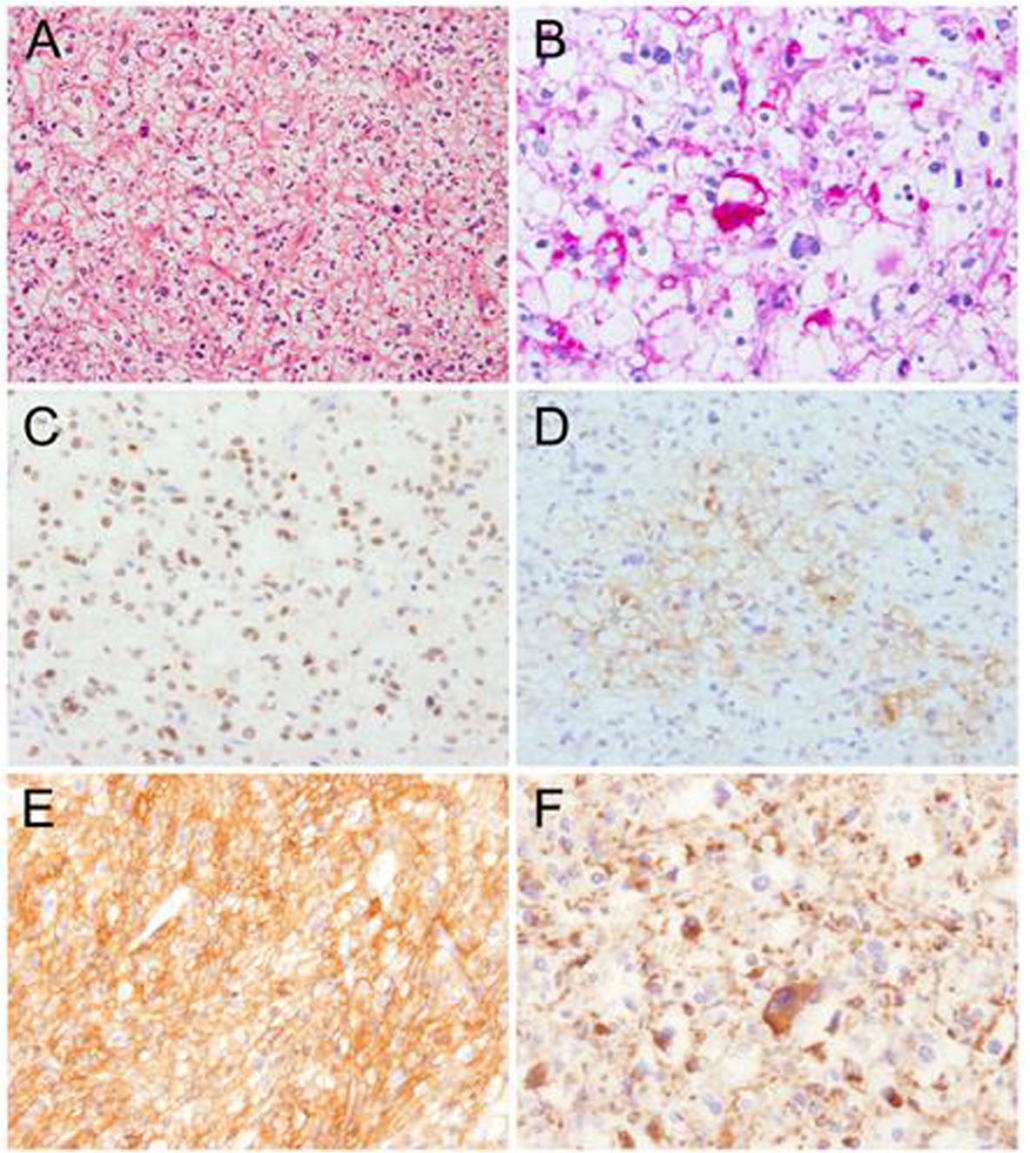
Pathologic findings of a pulmonary nodule in the left lower lobe. The nodule consisted of polygonal tumor cells with pleomorphic nuclei and clear cytoplasm (**a**, H&E). The PAS stain was intensively positive in cytoplasm of tumor cells (**b**). Immunohistochemistry revealed that nuclei of the tumor cells were diffusely positive for microphthalmia transcription factor (**c**), and their membrane was weakly positive for CD1a (**d**), strongly positive for CD10 (**e**), and focally positive for folliculin (**f**)

The two tumors, measuring 25 mm and 5 mm in diameter, respectively, were clearly demarcated from lung parenchyma. They showed identical histopathological findings and consisted of large polygonal cells with clear cytoplasm in a solid pattern (Fig. 3a). A meshwork-like vasculature separated these tumor cells, some of which were small with round nuclei but others of larger size contained bizarre nuclei without hyperchromaticity. The cytoplasm stained readily with periodic acid-Schiff (PAS) (Fig. 3b), but neither mitosis nor vascular invasion was seen. Although the preoperative diagnosis was clear cell carcinoma of undetermined origin, that designation was then corrected to clear cell “sugar” tumor (CCST) of the lung, belonging to a family of PEComa [19]. The results of immunohistochemistry are summarized in Table 1. Analysis revealed that the tumor cells were immunopositive for vimentin, microphthalmia transcription factor (Fig. 3c), S100, CD1a (Fig. 3d), CD10 (Fig. 3e), CD63, and folliculin (Fig. 3f), but immunonegative for cytokeratin, α-smooth muscle actin, HMB45, Melan-A, and PNL-2. Interestingly, immunopositivity for folliculin was stronger in the cytoplasm of large tumor cells with bizarre nuclei than in that of the small tumor cells with round nuclei (Fig. 3f). Additionally, there was no proliferation of lymphangioleiomyomatosis cells observed in the underlying lung parenchyma.

**Table 1:** Summary of immunoreactivity demonstrated in Birt-Hogg-Dubé syndrome-associated clear cell “sugar” tumor in the lung

Antibodies	Immunoreactivity
Cytokeratin	-
Vimentin	+ (Focally strong)
α-smooth muscle actin	-
HMB45	-
Melan-A	-
PNL-2	-
Microphthalmia transcription factor	+
S100	+ (Focally strong)
CD1a	+ (Focally weak)
CD10	+ (Focally strong)
CD63	+ (Diffusely weak)
folliclin	+ (Focally strong)

### Genetic diagnosis of BHD syndrome and LOH analysis

**Fig. 4:**
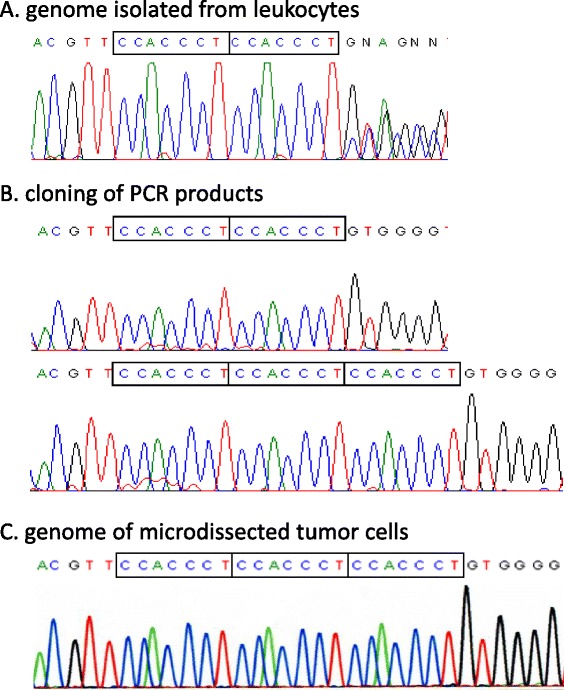
Mutation analysis of the *FLCN* gene. Sequencing of exon 12 of the *FLCN* gene demonstrated a superimposed sequence after the CCACCCT repeat when genomic DNA isolated from the index case’s peripheral blood leukocytes was used as a template (**a**). Cloning of the PCR product of exon 12 identified clones carrying the wild-type sequence and those carrying an insertion of 7 nucleotides (CCACCCT) (c.1347_1353dupCCACCCT), resulting in a frame shift and premature termination of protein translation (**b**). In contrast, only the mutated sequence with a 7-nucleotide insertion was demonstrated when genomic DNA isolated from microdissected tumor cells was utilized as a template (**c**)

Family history of pulmonary cysts/spontaneous PTX and renal tumors as well as characteristic radiologic features was strongly suggestive of BHD syndrome and prompted us to perform *FLCN* genetic testing. Mutation analysis identified a germline mutation on exon 12 of the *FLCN* gene, an insertion of 7 nucleotides (CCACCCT) (c. c.1347_1353dupCCACCCT) (Fig. 4). The expected result would be a frame shift and premature termination of protein translation [p.(Val452Profs*457)], thereby establishing the diagnosis of BHD syndrome.

**Fig. 5:**
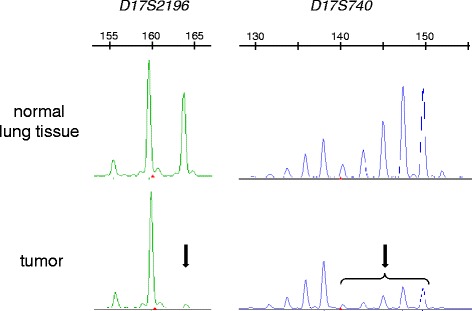
Results of loss-of-heterozygosity (LOH) analysis. Representative results of LOH analysis at *D17S2196* and *D17S740*. Genomic DNA was isolated from both normal lung tissue and tumor cells microdissected from lung pathological specimen. One of the alleles at both polymorphic markers nearly disappeared in tumor cells (*arrows*). The upper bar with numerals shows an electrophoretic position of DNA fragment size (*bases*)

CCST showed LOH at both *D17S740* and *D17S2196* (Fig. 5). Direct sequencing of exon 12 using genomic DNA obtained from the patient’s microdissected CCST cells (from the larger tumor) clearly revealed the loss of wild-type *FCLN* sequence (Fig. 4c), confirming the complete loss-of-function of the *FLCN* gene (Knudson’s 2-hit theory). In contrast, none of the microsatellite markers on two *TSC* loci (*TSC1* on chromosome 9q and *TSC2* on chromosome 16) manifested LOH (data not shown).

## Discussion

Our study reports, for the first time, a CCST of the lung in a patient with BHD syndrome. Additionally, no pulmonary neoplasm has been reported to date that was proved to have complete loss-of-function type *FLCN* mutation, hence meeting the Knudson’s 2-hit theory. CCST of the lung belongs to a family of PEComas that is characterized by perivascular epithelioid cell differentiation and immunopositivity for both myoid and melanocytic immunohistochemical markers; a representative of the latter is HMB45. These immunohistochemical profiles are adjunctively important to differentiate CCST from renal cell carcinoma. However, the existence of HMB45-negative PEComas was reported [20–23]. We examined the expression of four different markers of the melanocytic lineage (HMB45, Melan-A, PNL-2, and microphthalmia-associated transcription factor) in our patient and found that, of those markers, only microphthalmia-associated transcription factor was immunopositive. Accordingly, based on both morphological appearance and immunohistochemical features, we diagnosed this patient with HMB45-negative CCST of the lung.

This is the first example described that associates the development of CCST with functional loss of *FLCN*. Interestingly, this woman’s test results document a stronger immunopositivity for folliculin in the cytoplasm of large tumor cells with bizarre nuclei than in that of small tumor cells with round nuclei. Still unclear is whether the immunoreactivity we detected for polyclonal anti-folliculin antibody implies a causative role of mutated folliculin in the tumorigenesis of CCST or merely nonspecific binding. As is well-known, the development of PEComas is closely associated with dysregulated mTORC1 activity, which is often due to a functional loss of *TSC1* or *TSC2*. Although the function of folliculin is not fully understood, folliculin and mTORC1 are considered to be functionally related in the cellular signaling pathway; i.e., folliculin acts as a GTPase-activating-protein for RagC/D for the recruitment of mTORC1 to lysosomes, where mTORC1 exerts its function [11, 24]. Recently, a distinct subset of PEComas was reported to harbor a *TFE3* gene fusion [20]. Interestingly, these tumors are immunopositive for TFE3, but negative for markers of the melanocyte lineage, such as microphthalmia-associated transcription factor, Mart-1, and HMB45. The CCST in our patient showed no immunoreactivity for TFE3 (data not shown). Variability of immunostaining results regarding cell-lineage markers may be related to the uncertainty of CCST’s cellular origin [19]. However, the CCST described here showed membranous immunopositivity for CD1a, a useful marker for Langerhans cells, as Adachi et al. previously reported [25].

Clinically notable in the family of our patient are the many deaths from cancer in the maternal ancestors (Fig. 2). Multiple occurrences of neoplasms at various sites including uterus, thyroid, vocal cord, and the lung in the index case are also remarkable. Although *FLCN* is considered to function as a tumor-suppressor gene, the established propensity for cancer in BHD syndrome so far is limited to the renal neoplasms [26–28]; whether BHD syndrome confers the risk of developing colorectal cancer remains conjectural [29]. We previously reported that the *de novo FLCN* mutation occurred in descendents of patients with familial adenomatous polyposis [30], a disease caused by germline mutation of the *APC* gene. Historically, BHD syndrome was first identified in a family who developed hereditary medullary carcinoma of the thyroid, a disease now recognized as a tumor-suppressor gene syndrome caused by a germline *RET* mutation [2]. In this context, the present family may represent a *de novo FLCN* mutation that emerged in the patient’s mother and grandmother who may have descended from a family with an unknown cancer propensity.

## Conclusions

To our knowledge, this is the first report of CCST of the lung in a patient with BHD syndrome and may suggest that folliculin and mTORC1 are closely associated with the tumorigenesis of this rare affliction.

## Abbreviations

BHDS: Birt-Hogg-Dubé syndrome
CCST: Clear cell sugar tumor
CT: Computer-assisted tomography
FLCN: Folliculin
H&E: Hematoxylin and eosin
LOH: Loss of heterozygosity
mTOR: Mechanistic target of rapamycin
mTORC1: Mechanistic target of rapamycin complex-1
PAS: Periodic acid-Schiff
PEComa: Perivascular epithelioid cell tumors
PTX: Pneumothorax
TSC: Tuberous sclerosis complex
α-SMA: α-smooth muscle actin
